# Phenotypic plasticity influences the success of clonal propagation in industrial pharmaceutical *Cannabis sativa*

**DOI:** 10.1371/journal.pone.0213434

**Published:** 2019-03-18

**Authors:** Lesley G. Campbell, Steve G. U. Naraine, Jaimie Dusfresne

**Affiliations:** Department of Chemistry and Biology, Ryerson University, Toronto, Canada; College of Agricultural Sciences, UNITED STATES

## Abstract

The burgeoning cannabis market requires evidence-based science such that farmers can quickly and efficiently generate new plants. In part, horticultural operations are limited by the success of cloning procedures. Here, we measured the role of environmental conditions and cultivar identity on the success of generating long branch material with many meristems in planting stock (mothers) and in rooting success of stem-derived clones. To evaluate the influence of lighting treatments on the optimal production of branching mothers, four lighting conditions (Fluorescent High Output T5s [T5], Metal halide lamps [MH], Plasma lamps [PL], or Metal halide lamps augmented with far red LED lights [MH+FR]) were applied to two cultivars of container grown plants (*Cannabis sativa* L. ‘Bubba Kush’, ‘Ghost Train Haze’) grown in peat-based organic substrates in mylar grow tents. To evaluate the influence of lighting, cutting tool (secateurs or scalpels), and stem wounding (present/absent) on optimal rooting of stems, three lighting conditions (Fluorescent T8s, T5, PL) were applied to three cultivars of peat pellet grown plants (*C*. *sativa* L. ‘Bubba Kush’, ‘Ghost Train Haze’, ‘Headband’). Mothers grown under T5 and MH (vs MH+FR) produced ~30% more meristems. However, growing mothers under MH+FR were 19% taller than mothers under T5, with ~25% longer internodes on dominant stems than plants under any other lighting condition. Canopies were denser under T5 because petiole length was ~30% shorter under T5 and fan leaves were longer and narrower under MH+FR and MH+FR and PL, respectively, than under other lighting conditions. Cultivar Ghost Train Haze stems rooted most frequently and most quickly. Wounded stems were 162% more likely to root than unwounded stems and rooted 1.5 days earlier. Our results will guide producers attempting to maximize the rate of clone production in licensed facilities; although results may differ among cultivars, where cultivars differed in their average phenotype as mother plants, and their propensity to root from cuttings, and the speed with which they produced those roots.

## Introduction

*Cannabis sativa* is an annual crop that has been widely cultivated for its fiber, nutritional content, and medicinal purposes [1,2] and is experiencing rapid growth in the industrial-scale production and consumption of the plant. A group of terpenes called cannabinoids largely influence the psychoactive effects of the plant and selective breeding has produced high-potency marijuana cultivars that serve to supply the majority of cannabinoids consumed globally. Cannabis is estimated to be a $22.6B industry in Canada [3], and the demand for safe, consistent, high-quality Cannabis has outstripped supply following legalization. Knowledge of the environmental conditions and horticulture practices that maximize the Cannabis production is limited, with few publications describing evidenced-based practices, although we review available knowledge next. This emerging market requires evidence-based science, such that cannabis producers can quickly and efficiently generate new plants. The rate at which new plants are generated is, in part, limited by the success of cloning procedures. To produce clones, cultivators harvest stems from mother plants and cut these stems into short sections (clones) with each section possessing meristems on at least three nodes: one meristem will produce new roots, the other two may produce leaves. Therefore, the success of clonal propagation within industrial production facilities is a key determinant in the efficiency of operations. Here, we explored the influence of cultivar and environmental conditions on stem growth of mother plants and on rooting success of cuttings subsequently derived from the mother plants.

Mother plant size and stem architecture determines the number of clones that can be harvested from a plant. One of the most influential environmental conditions to affect cannabis production is lighting [4,5], since light radiation is a key environmental signal that regulates plant growth [6], morphology [7], and secondary metabolite chemistry [8], and particular wavelengths of light have very prescriptive responses. For instance, many plants increase internode length as a response to intercepting increased far-red [e.g., 9–11], which could influence the number of clones that a mother could produce. In contrast, metal halide lights tend to radiate a spectrum which is expected to reduce internode length but may also limit growth. Finally, plasma lights exhibit more uniform photosynthetic photon flux and better replicate solar spectra, and thus may promote more growth indoors [12]. Modifications to lighting may alter the physical architecture of mother plants, allowing a single mother to produce more clones. When restricted to growing indoors, as in the Canadian medical marihuana industry, growers must provide supplemental light to grow plants and manipulate photoperiods [4,5,13]. However, there is a dizzying variety of lighting technologies available, with very sparse peer-reviewed data comparing systems and their impact on cannabis clone growth, development and yield. In particular, there is little evidenced-based research available on the sensitivity of cannabis mother plants to lighting environments and the consequences of lighting for clonal propagation, especially the influence of light source on diverse cultivars grown and across life stages.

A number of environmental conditions can influence the growth and rooting speed of vegetative propagation including humidity [14], temperature [15,16], cutting length, stem thickness [17,18], season [16,18], and the age and health of mother plants [16,19]. Yet, there is little published data describing the effect of light, cutting method, or spatial arrangement of cuttings on the success of those cuttings. By modifying the conditions under which clones are collected and grown, we could increase the number of clones that successful produce roots and the speed at which they do so. Basal stem wounding when combined with applications of the auxin IBA (1H-indole- 3-butanoic acid) has been shown to encourage rooting in difficult to propagate plant populations [14,15,20,21]. Exposure of vascular cambium and secondary xylem (i.e., wounding) to the rooting environment can be beneficial by inducing hormonal changes in plant tissues capable of producing lateral roots [22]. In the propagation of *Juniperus osteosperma* or *Abies fraseri*, wounding apparently enhances rooting when also exposed to auxins (e.g., indole-3-butyric acid, IBA [21]). Importantly, wounding alone does not increase rooting ability [16], implying that wounding largely acts to make pericycle cells (which initiate lateral root formation) more accessible to exogenous applications of auxin. Further, wounding, in concert with auxin application, has expressed varied results in dicots. Jojoba (*Simmond siachinensis*) stem cuttings show roots emerging from the entire wound rather than just the base, although there is no increase in overall success or time to root [20]. Alternatively, *Colutea istria* displays increased rooting success and root number when wounded [14]. Some cannabis growers suggest wounding may be a useful method for enhancing rooting speed in the high-throughput production of clones.

Finally, managing water stress in soft stem cuttings is essential to encouraging rooting of clones. Previous studies have revealed that optimal relative humidity conditions for rooting are species-specific requirements [e.g., 14,23]. If wounding is not imposed, a 45° cut maximizes the contact between tissue surface area with the rooting medium. However, if a tool damages the stem’s vascular system during the process of making the cut, then a clone will likely experience water stress regardless of other environmental conditions [24]. Moreover, vascular tissue may be occluded by microorganisms that are present on the stem or transferred from cutting surface during the cloning process [25,26]. Notably, we did not encounter studies comparing the efficacy of cutting tools in the primary literature. We chose to compare two cutting methods to clonally propagate clones from mother plants. Sharp scalpels may cause less tissue damage than pruning shears, in addition to being easier to clean. In contrast, pruning shears require less labor and are safer to use.

Our objectives were to identify optimal lighting environments for the productive of mothers and to assess the ideal method for producing rooting stems (clones). We asked:

1. How does lighting condition (fluorescent high output T5s, 1000W metal halide / high pressure sodium lights, metal halide/high pressure sodium lamps augmented with far red LED lights, or plasma lamps) and genotype (Bubba Kush or Ghost Train Haze) influence the number and size of clones that a mother plants generate? and,
2. Does rooting success of stem cuttings vary among genotypes (Bubba Kush, Ghost Train Haze, Headband) and lighting environments (fluorescent T8s, fluorescent high output T5s, plasma lights)?

## Methods and methods

### Plant genotypes

Only female plants were used in the following two experiments (the Mother Experiment and the Cloning Experiment). Two cultivars, Bubba Kush and Ghost Train Haze were included in both experiments, so that we had the opportunity to measure the response of two genetically and phenotypically distinct populations to variation in lighting conditions. Bubba Kush possessed high THC:CBD ratio (THC = 13.1%, CBD <0.05%) and typically flowered in 63 days. Ghost Train Haze also exhibited a high THC:CBD ratio (THC = 25%, CBD = 0.07%) and typically flowered in 70–84 days. A third cultivar was included in the cloning experiment only: Headband also exhibited a high THC:CBD ratio (THC: 17.6%, CBD: 0) and an intermediate flowering phenology.

### Plant culture

Seeds were planted on November 27, 2013 in 25.4cm x 50.8cm black plastic seedling trays with 72-cell inserts and later transplanted (December, 2013) into square nursery pots (8.89cm x 8.89cm x 10.16cm). These plants were transplanted into 7.6L pots in late January, 2014 and 18.9L pots in early April, 2014 to create a population of potential mothers. All plants were watered and fertilized according to the Tweed standard operating procedures (Tweed Inc., City, Canada) and grown under 6400K T5 fluorescent lights until the experiments began. Cultivars Ghost Train Haze and Headband mothers were grown under T5 lighting prior to cloning, while Bubba Kush was maintained under 1000W metal halide lamps.

For the cloning experiment, all clones were taken on the same day, December 10th, 2014. A single mother plant of each variety produced 244 Ghost Train Haze clones and 288 Headband clones, ensuring genetic homogeneity of experimental plants. A total of 190 Bubba Kush clones were taken from three mother plants, one grown from seed for 11 months and two secondary mothers (i.e., clones of the first mother), both grown for about 7 months.

Branches, 20 cm long, were initially cut from mothers with a pair of pruning shears and were placed in a solution of 500 ml water and 5 ml of 5% hydrogen peroxide until the application of treatments described below. Cut clones were stripped of lower fan leaves using pruning shears, and then were re-cut at a 45° angle to a 15cm length. Pruning shears and scalpels were replaced regularly to ensure they remained sharp. The bottom of each clone stem was then dipped in #1 Stim Root (Master PlantProd Inc., Brampton, ON, Canada), inserted in a Grow-Tech Flexi-Plug® with the stem tip approximately 1.5 cm from the plug base (Quick Plug, South Portland, ME, USA) and placed into a seedling tray. The trays were covered with 7” Aztec humidity domes with closed vents, and were placed in their appropriate experimental block on a Hydrofarm seedling heat mat (Hydrofarm, Petaluma, CA, USA) connected to a Jump Start Digital Temperature Controller for Heat mats (Hydrofarm) set to a temperature of 26⁰C. In the production facility, clones generally produced roots between 5 to 35 days following cloning, depending on the genotype and environmental conditions.

To create isolated blocks, we used independently HEPA ventilated 2.41m x 1.22m X 2.01m silver mylar grow tents under positive atmospheric pressure, limiting potential aerial contamination. Each tent contained four 36-cell cloning trays (53.3cm x 27.9cm x 7.6cm) that were paired and spaced two feet apart in the center of a tent for a total of 144 possible clones. Clones were randomly and blindly assigned to a treatment combination, tray, and location within trays to minimize positional effects within a block and limit experimenter bias. Clones were watered as needed with a solution of 76L water, a company-specific 76L nutrient solution, and 340 ml hydrogen peroxide at a pH of ~5.8, and an EC of ~1.20 mS/cm. On day three, the humidity dome vents were opened. On day six, the domes were removed for several hours a day, and entirely removed on day 10. Daily air temperature and relative humidity of three locations within the tent were recorded and exhaust fans were used to maintain similar environmental conditions across tents. Additionally, external temperature probes were placed in the base of two clone trays per tent to measure the approximate root zone temperature.

### Treatments imposed during the mother experiment

**Fig 1:**
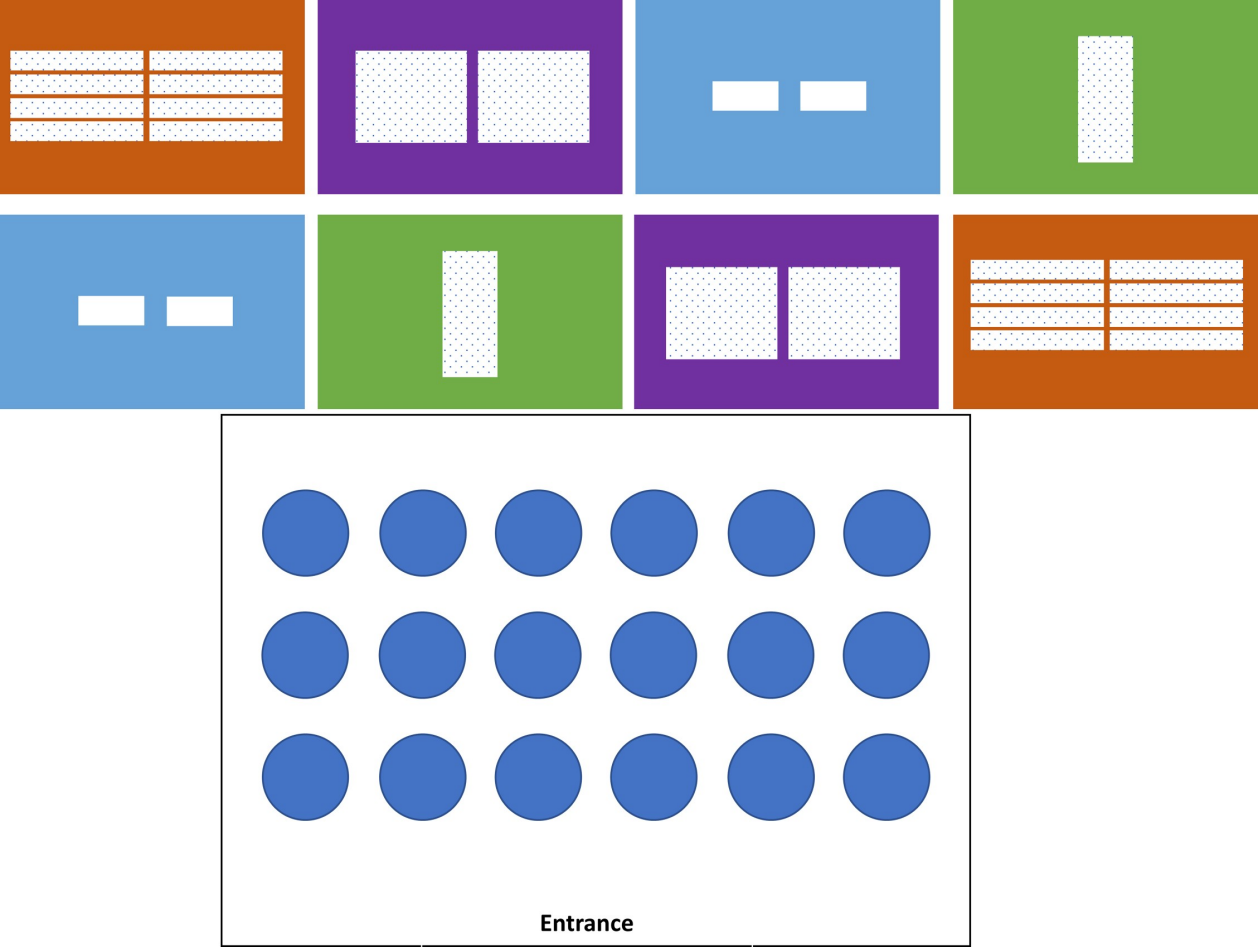
Schematic of experiments to quantify the effect of lighting and genotype used on the growth and morphology of Cannabis mothers. A) Blue, red, purple and green rectangles represent the plasma, T5, Metal Halide and Metal Halide tents, respectively. White squares within each tent represent the approximate size and placement of lamp fixtures. B) Plant layout within each experimental tent.

To determine the influence of lighting environment and cultivar on the production of branch lengths that were appropriate for producing clones from mother plants, we designed a complete randomized block experiment where plants of two cultivars (Bubba Kush, Ghost Train Haze) were randomly assigned to tents that exposed plants to one of four lighting treatments. We compared the growth of six vegetative plants per genotype under one of four lighting treatments (or a total of 144 mother plants): fluorescent high output T5s (6400K), 1000W metal halide / high pressure sodium lights (1000W), metal halide/high pressure sodium lamps augmented with far red LED lights (730-735nm Far Red 50W LED Lighting), or plasma lamps (300W) (Fig 1). The photon flux within a tent was measured at multiple sites within a block and was standardized to within 10 umol/m^2^/s across all replicates (~104 to 110 umol/m^2^/s). As per industry practice, we used a 24-hour photoperiod and raised the lights as plants grew to maintain a standard height (120cm) of the lights above plants for the duration of the experiment.

### Treatments imposed during the cloning experiment

**Fig 2:**
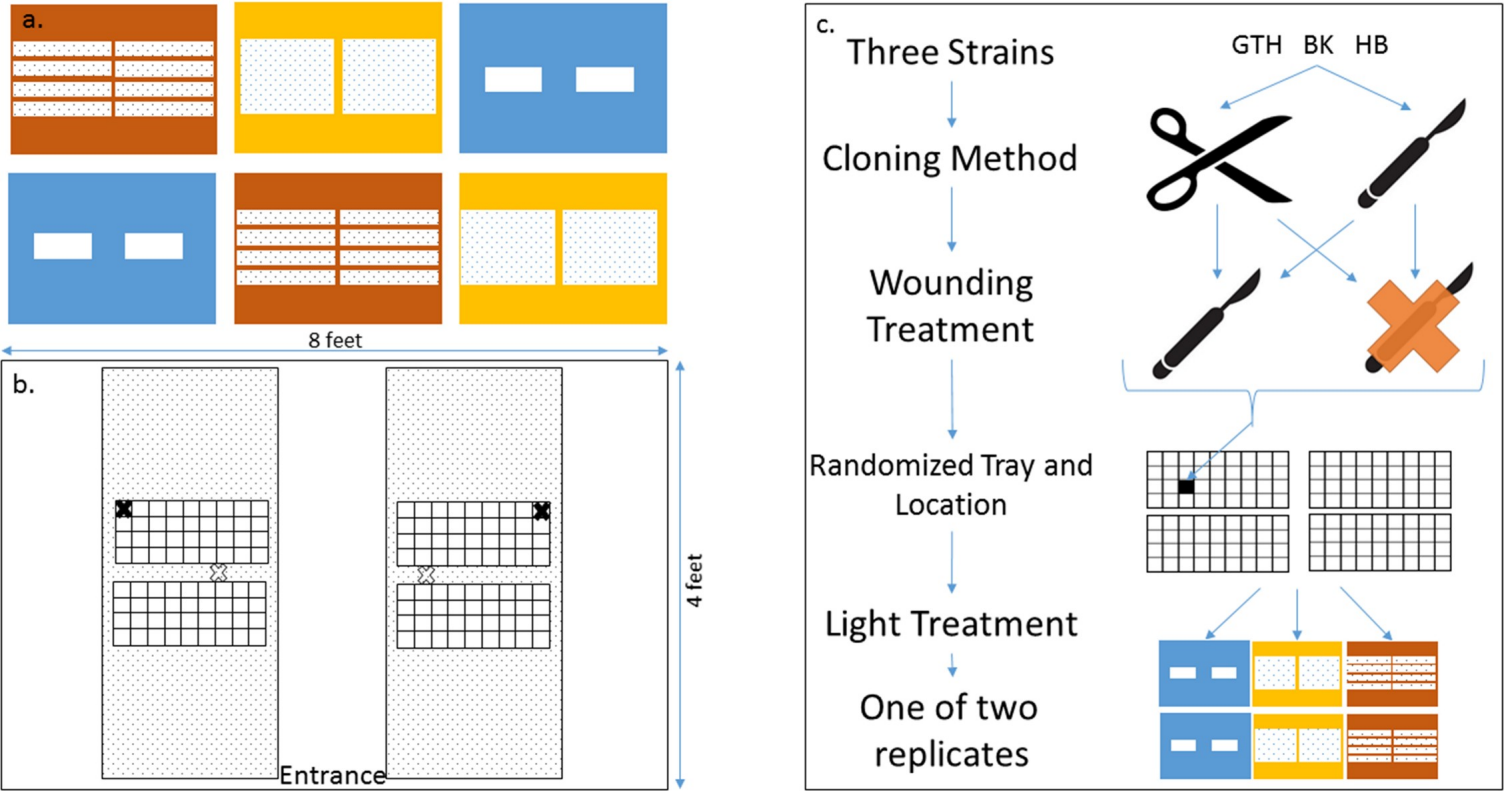
Schematic of experiments to quantify the effect of lighting, stem scarring, and tool used on the rooting probability and timing of Cannabis clones. A) Blue, red, and yellow rectangles represent the plasma, T5, and T8 tents respectively. White squares within each tent represent the approximate size and placement of lamp fixtures. B) Tray layout within each experimental tent; black X—air temperature/humidity probe, white X- seedling heating mat thermostat probe C) Experimental procedure. Cuttings were taken using either secateurs or a scalpel and were randomly allocated to a tray position in a lighting treatments replicate.

For the experiment that evaluated successful methods for creating clones, we used a randomized block design where cutting tool, stem wounding, lighting, and genotype were main effects with two replicates per combination (Fig 2), with ~12 stems per genotype by cutting tool by wounding treatment combination or 836 clones involved. To determine if the cutting tool influenced rooting success, branches were cut into clones (that were 15cm long) with either a disposable scalpel or with pruning shears. To determine whether clones that had experienced wounding were more likely to produce roots, half of the clones were wounded by removing epidermal tissue from the bottom 5cm of a clone’s stem by scraping a scalpel parallel to the stem surface. Three lighting treatments were considered in our design. We compared the rooting behaviour of clones under Fluorescent T8s (3200K), Fluorescent High Output T5s (6400K), and 300W plasma lights. The photon flux within a tent was measured at multiple sites within a block and was standardized to within 10 umol/m^2^/s across all replicates (~104 to 110 umol/m^2^/s). As per industry practice, we used a 24-hour photoperiod. Since the height of the clones did not change by more than several centimeters over the course of the experiment, the lights remained the same distance from the clones for the duration of the experiment.

### Data collection

In the mother experiment, before the experiment started and weekly thereafter, we assessed a variety of morphological characteristics of each plant prior to the start of the experiment and at the end of the experiment (week 4). The amount of nutrients leaching from each plant (EC) and the soil pH were measured. For leachate testing, we watered each plant with 1L of water, waited an hour and added 1.5L of water, collecting all of the leachate from the second watering using a funnel and a clean container. We then used an EC/pH meter (Hanna Instruments 98129 pH/Conductivity/TDS combo pen) to measure the characteristics of the leachate. All equipment was rinsed using pure water before re-use.

On each plant, we measured key morphological features to predict clonal yield and robustness of plants for the mother experiment (S1 Fig). We measured the height of each plant from the first node to the tallest apical meristem and the stem diameter (one measurement at the base of the plant and one measurement at 10cm above the base of the plant; VWR calipers #36934–154, accuracy: +/- 0.2mm, resolution: 0.1mm). We counted the number of apical and lateral meristems. We measured the length of all lateral branches derived from the shortest and the tallest upright branches. On each individual, we measured the length of all internodes on the tallest and shortest upright branches. Because leaf area is both responsive to light quality and important for photosynthetic assimilation, we also measured the morphological consequences of light on leaf shape. On three haphazardly selected petioles, the length of the petiole was assessed. We measured the width of the three largest fan leaf blades at the widest point, the width of the widest point of leaflets of these same three leaves.

In the cloning experiment, to determine date of rooting, Flexi-Plugs were checked for emergent roots from day 7 to 26 (S2 Fig). Rooted clones were marked, and left untouched for the remaining duration of the experiment.

### Data analyses

To determine whether lighting treatment or cultivar (and their interaction) influenced the branch lengths, we ran a multivariate ANOVA in SPSS (IBM SPSS Statistics for Citrix, Version 24.0. Armonk, NY: IBM Corp.) on 16 measurements collected in the fourth week of the mother experiment (EC, pH, number of meristems, number of apical meristems, number of lateral meristems, plant height, average length of internodes on the tallest and shortest stem, petiole length, fan leaf width, length and width of the largest leaflet, stem diameter at the base of the plant and at 10 cm above the soil surface, length of the longest lateral branch on the tallest stem, length of the longest lateral branch on the shortest stem. Before MANOVA analysis, we z-transformed all data (S1 Dataset; transformed data are available from OSF database at: https://osf.io/qdnkh/?view_only=237af5bf977148b4b60e599caec74159). If a main effect was significant, *a priori* contrasts were then performed to determine differences among lighting conditions (all pair-wise comparisons) and cultivars.

Before running analyses on clones, we removed two plants with broken stems from the cloning dataset, because this early experience may affect their likelihood of rooting. To determine whether lighting, cultivar, position within tray, wounding, or cutting tool would affect the likelihood of rooting, we ran a nested mixed model logistic regression using the glmer function in lme4 in R [27] where the binary response variable was rooting status (yes or no, see S1 Code). After running the full model, several main effects (and their interactions) with non-significant effects were removed from the model (light, cutting tool, position within tray, number of fan leaves). The final model was: Rooting status ~ Intercept + Cultivar + Wound +Cultivar × Wound + Tray within Tent + Tent within Light + Error.

With the 520 clones that rooted, we then assessed the influence of lighting, genotype, wounding or cutting tool on the timing of rooting using a GLM ANOVA in SPSS, controlling for the number of fan leaves. Again, the full model failed due to lack of degrees of freedom. After removing the genotype x lighting x wounding x cutting tool interaction, there were enough degrees of freedom for the model to perform as expected. All non-significant interactions that were subsequently detected were also removed from the model; we found no significant effect of several main effects (lighting, cutting tool, number of fan leaves) and removed them (and their interactions) from the ANOVA.

## Results

### Which lighting condition maximizes the number and size of clones that a mother generated?

**Fig 3:**
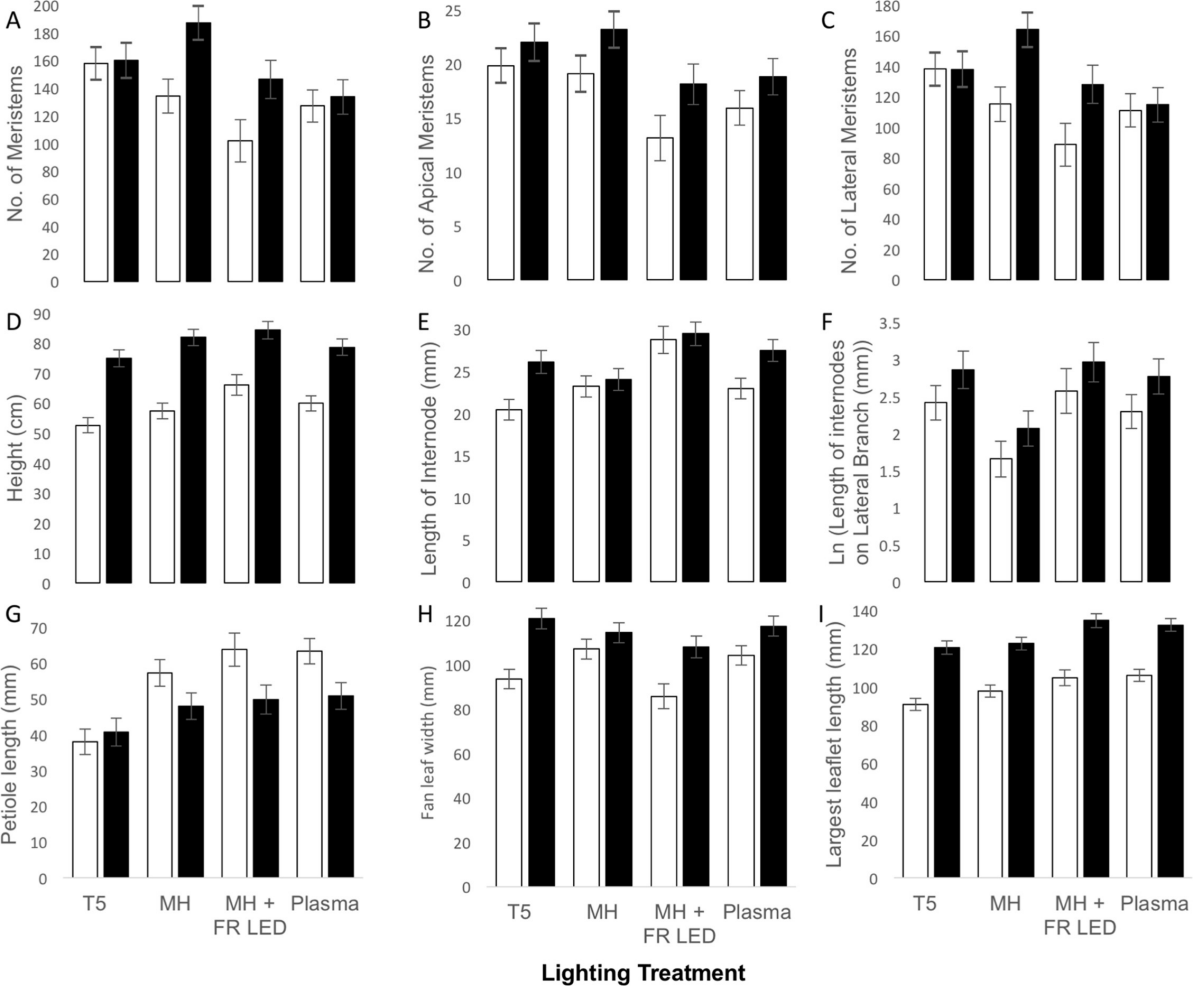
Comparison of nine key traits to predict clonal yield and robustness of “mother” *Cannabis sativa* plants grown under one of four lighting treatments. Lighting treatments included T5 fluorescent bulbs, metal halide lamps (MH), metal halide lamps augmented with far red LEDs (MH + FR LED), and Plasma lamps. Averages represent trait means of 48 mother plants from two cultivars (White = Bubba Kush, Black = Ghost Train Haze); error bars represent the SE of the mean.

By the fourth week of the experiment, the mother phenotype differentially responded to lighting conditions (MANOVA: F_39,192_ = 3.68, P<0.001) and genotype (MANOVA: F_13,62_ = 31.25, P<0.001) and their interaction was marginally significant (MANOVA: F_39,192_ = 1.34, P = 0.10). Specifically, plants grown under T5 and metal halide lamps produced more meristems (both apical and lateral) than plants grown under metal halide lamps augmented with far red spectra (See Fig 3; See Table 1 for tests of between-subject effects of the MANOVA described earlier). However, plants grown under metal halide lamps augmented with far-red spectra grew taller than plants grown under T5 bulbs and longer internodes on dominant stems than plants grown under any other lighting condition. Plants grown under metal halide lamps produced significantly shorter internodes on lateral branches than plants grown under other lighting treatments. Leaf morphology also changed in response to lighting conditions. Petiole length was ~30% shorter under T5 lamps, making the canopy denser, than under other lighting conditions. Further, fan leaves were narrower under metal halide lamps augmented with far-red spectra. Finally, leaflets were longest under metal halide lamps augmented with far-red spectra and plasma lights.

**Table 1:** A comparison of key traits to predict clonal yield and robustness of “mother” *Cannabis sativa* plants grown under one of four lighting treatments from one of two cultivars (genotype).

Dependent Variable (transformed to z-scores)	R^2^	Lighting (L)		Genotype (G)		L x G	
		df	F	df	F	df	F
No. Meristems	0.26	3,75	**4.34**	1,75	**8.55**	3,75	2.13^+^
No. Apical Meristems	0.22	3,75	**4.52**	1,75	**8.10**	3,75	0.24
No. Lateral Meristems	0.25	3,75	**3.84**	1,75	**7.65**	3,75	2.34^+^
Height	0.65	3,75	**5.16**	1,75	**112.12**	3,75	0.58
Avg. Length of Internodes on Tallest Stem	0.34	3,75	**6.89**	1,75	**9.51**	3,75	1.82
Avg. Length of Internodes on Shortest Stem	0.09	3,75	1.79	1,75	1.20	3,75	0.15
Avg. Length of Internodes on Lateral Branches on Tallest Stem	0.09	3,75	1.91	1,75	0.13	3,75	0.52
Avg. Length of Internodes on Lateral Branches on Shortest Stem	0.13	3,75	1.89	1,75	**5.01**	3,75	0.07
Petiole Length	0.37	3,75	**9.78**	1,75	**9.15**	3,75	1.95
Width of Fan leaf	0.35	3,75	**3.34**	1,75	**27.63**	3,75	1.90
Length of Largest Leaflet	0.69	3,75	**8.13**	1,75	**131.37**	3,75	0.27
Width of Largest Leaflet	0.17	3,75	2.44^+^	1,75	3.37^+^	3,75	1.43
Stem diameter at base	0.10	3,75	0.86	1,75	0.50	3,75	1.65
Stem diameter 10 cm above base	0.10	3,75	1.22	1,75	3.75^+^	3,75	0.10

We performed a multivariate ANOVA for each trait (transformed z-scores) for two cultivars (Bubba Kush, Ghost Train Haze). Plants were exposed to one of four lighting environments: Lighting treatments included T5 fluorescent bulbs, metal halide lamps (MH), metal halide lamps augmented with far red LEDs (MH + FR LED), and Plasma lamps. F-statistics are presented to indicate significant differences: not bold type, P > 0.10

+, P < 0.10; bolded type, P < 0.05.

Predictably, cultivars differed in their plant architecture (Table 1, Fig 3). In particular, Ghost Train Haze produced 20% more meristems (apical and lateral), grew 35.5% taller, and exhibited 12% longer internodes than Bubba Kush. Further, Ghost Train Haze exhibited a denser canopy with 15% shorter petioles, 18% wider and 28% longer leaves than Bubba Kush. We did not detect any significant genotype by lighting environment interactions for any measured trait (Table 1).

### Does rooting vary among genotypes and environments?

**Fig 4:**
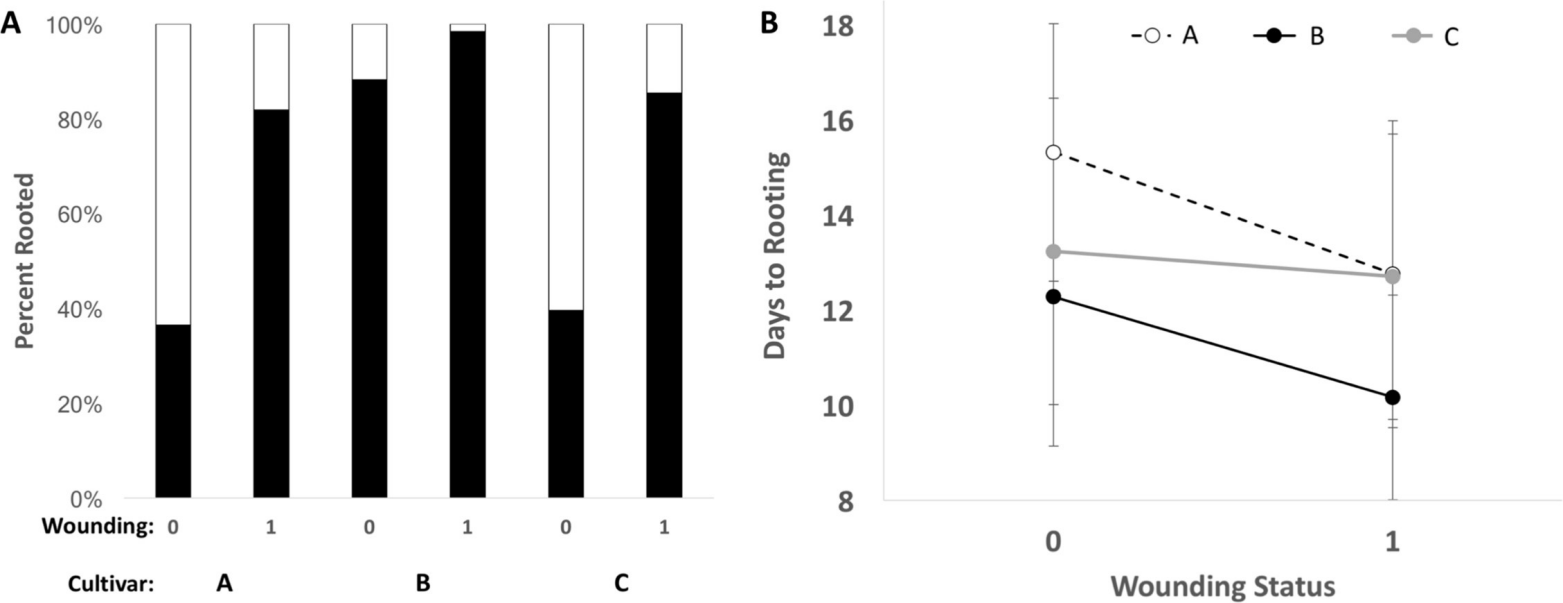
Response of clonal rooting to environmental and genetic context. A) Frequency of cuttings that produced roots for three cultivars (A: Bubba Kush, B: Ghost Train Haze, C: Headband), depending on whether stem cuttings were wounded (1) or not (0). B) Average number of days until stem cuttings produced roots for three cultivars and experimental wounding.

Cultivars differed significantly in the tendency for cut stems to produce roots and the speed with which they rooted (Table 2). Of the cultivars we tested, Ghost Train Haze rooted most frequently and most quickly (Fig 4). Stem wounding improved rooting success (Table 2) such that wounded plants were 162% more likely to root than unwounded stems and rooted 1.5 days earlier (Fig 4). There was no significant interaction of cultivar and wounding for the tendency to root (Table 2A); however, there was a significant interaction for days to rooting (Table 2B). Specifically, days to rooting for Headband was unresponsive to wounding treatment, whereas days to rooting was shortened for Bubba Kush and Ghost Train Haze when wounding was applied (Fig 4).

**Table 2:** Logistic regression analysis of A) rooting success and B) date of rooting across three cultivars (Bubba Kush, Ghost Train Haze, Headband) that either experienced stem wounding or not during the generation of clones.

	Factor	df	F-value	P-value
A	Cultivar (C)	2,2	25.26	0.038
	Wounding (W)	1,2	102.4048	0.010
	C x W	2,715	0.0474	0.95
B	Cultivar (C)	2,2	42.9801	0.023
	Wounding (W)	1,2	46.6819	0.021
	C x W	2,512	5.5421	0.0042

Cultivar interactions with Wounding were included to evaluate the degree to which genotype effects were context-dependent. The Generalized linear mixed model fit by maximum likelihood also accounted for the variance associated with tray nested within tent.

### Substrate EC and pH

EC did not change significantly among genotypes nor lighting conditions (nor their interaction; Table 1). Similarly, pH did not change significantly among genotypes nor the interaction of lighting and genotype but there were significant differences among lighting treatments in the fourth week of data measurements (end of the mother experiment) for pH (Table 1). pH was ~0.48 units lower in pots grown under metal halide lamps that supplemented with Far Red LED lighting than in pots grown under T5 lights.

## Discussion

Adjustments in lighting environments for mother plants generally produced either plants with many more meristems (T5 and MH, ~160 meristems) but short internode lengths (~23 mm) or fewer meristems (~124 meristems) with longer internodes (MH+FR; ~29mm), revealing a life-history trade-off that will influence production of clones. Notably, one cultivar (i.e., Ghost Train Haze) would be easier to use as a source of clones because it produces more meristems and longer internodes and stem cuttings were more likely to root quickly than the other cultivars. Importantly, we did not detect any reaction norms (because there were no significant genotype by lighting environment interactions) suggesting that these *C*. *sativa* genotypes may already be selected for “stable” cultivated genotypes. Finally, the production of adventitious roots in stem cuttings appears to be positively influenced by stem wounding but not influenced by lighting condition or cutting tool. These results suggest that clonal propagation of cannabis may be increased by wounding stem cuttings and may be influenced by diverse lighting conditions for mother plants, depending upon the desired morphological outcome. Specifically, if the grower is aiming for many meristems on mother plants, we recommend using either T5 fluorescent or metal halide lighting, whereas if a grower’s goal is long internodes, then we recommend using metal halide lighting augmented with far red LEDs.

Within a cannabis operation, mother plants serve as a source of stem cuttings to propagate the next crop of harvested plants. As such, an ideal plant and cultivar would possess large quantities of meristems and reasonably long internodes (~40–80 mm) such that a single cutting would be composed of three nodes and two internodes of ~75 mm each. Finally, because leaf area influences photosynthetic assimilation rates, the leaves of an ideal mother plant would be relatively unresponsive to shifts in light. As predicted based on other studies of photomorphogenic responses [summarized in 28], the four light spectra had a strong influence on plant architecture but revealed a trade-off between number of meristems and length of internodes. Under far red LED lighting, internodes were stretched to 29 mm and ranged between 5–93 mm, depending on the plant, genotype, and lighting condition. Under MH+FR, ~5 internodes (6 nodes) would be needed to create a stem cutting 15 cm long whereas under T5s, 6 internodes and 7 nodes would create a 15cm stem cutting. Therefore, under T5 lighting, plants would create 22 stem cuttings, whereas under MH+FR lighting, a plant would produce 20 stem cuttings that were 15 cm long, if almost the entire plant was useable. Since the difference among lighting conditions is negligible for the volume of clones produced, selection of lighting is perhaps best decided by a grower’s preference of clone morphology, either relatively long or short internodes. One of this study’s intentions was to elongate the internodes (length of stem between leaves/lateral branches), and although changing lighting conditions to metal halide augmented with Far Red LEDs (relative to all other lighting treatments) lengthened internodes in statistically significant ways, the increase is still perhaps industrially insignificant ways given the trade-off detected.

It is difficult to attribute plant morphogenic responses to specific physiological pathways mediated by the light environment in this experiment, because the light spectra used differed in many ways. However, there has been extensive research on two conspicuous characteristics that differ among light environments. Although they have been reviewed elsewhere [e.g., 29], we briefly mention them here. First, plants grown under altered red to far red light ratios are generally taller with longer petioles, and invest relatively more dry biomass in the stem, at the cost of partitioning dry biomass to the leaves [30]. Second, sun-leaves (with smaller leaf area and a high photosynthetic capacity) develop when exposed to a greater blue light fraction, or a higher absolute amount of blue light [31–33]. However, it is difficult to draw reliable conclusions on the mechanisms underlying the responses of the plants grown under the various spectra used in this study because of the interaction of blue light fraction, R:FR ratio, and other differences in the spectrum.

More dramatically, cultivar selection will influence the rate of clone production, since genotype had such a significant impact on both the number of stem cuttings available and their rate of rooting. High demographic recruitment rates in other, naturally clonal species are maintained by both high rates of clonal propagation and low variance among genotypes in clonal recruitment [34]. Growth rates commonly differ among genotypes in many plant species [35,36]. Importantly, many plant species have shown genotypic differences in plant architecture and physiology in response to environmental variation, including visible spectra [4,37]. Thus, choosing cultivars that show aggressive growth rates and that tend to naturally have longer internodes may improve yield of clones from mothers. However, this is rarely the single most important consideration for cultivar selection in a licensed facility. Futher, we failed to find any significant genotype by environment effects for any trait, which taken together suggests that for these cultivars, a genotype’s ability to exhibit plasticity in growth form in the face of different environmental conditions is not genetically determined [38]. This may be a result of repeated informal selection by growers for “stable” genotypes.

Like several other crops where one sex is economically important (e.g., jojoba, fibre hemp, asparagus), it is important to be able to quickly clone plants to increase productivity and reduce variability in crop performance [20,39,40] and strategies for improving the efficiency of clonal propagation have been studied for a century [41]. While clonal propagation is widely used, specific methods used vary considerably, along with degree of success. Several studies report both increased rooting and number of roots, when plant stems are wounded, and our results are consistent with this [20,42]. *Cannabis sativa* has been regularly vegetatively propagated [8,43] and various cultural strategies improve rooting success of stem segments including the use of IBA. Although the mechanisms behind how wounding would serve to increase adventitious root formation is unknown in *C*. *sativa*, it appears as though wounding can result in the release of polyphenol oxidase or jasmonic acid in other plants, chemicals that support rapid root growth (sometimes via shuttling sugars, for instance to the sites of growth [44,45]. Similar to a recent study which tested the effect of leaf number [43], we also noted no significant effect of this variable. However, our study adds wounding as a successful strategy to the horticultural toolbox of a Cannabis propagator.

In summary, we found that some cultivars possess more traits that make it easier for harvesting stem cuttings and light can augment their plant architecture for the purposes of clonal propagation in *C*. *sativa*. These differences were expressed as changes in the number of meristems and internodes. Further, our data is the first the reveal the tendency for cannabis stem cuttings to produce adventitious roots is driven both by genotype and stem wounding practices.

## Supporting information

S1 Dataset Z-transformed data from the mother and cloning experiments in a file named supplementary data. (XLSX)

S1 Fig Detailed description of the data collected from mother plants. (DOCX)

S2 Fig Detailed description of the data collected from stem cuttings. (DOCX)

S1 Code R code for binomial analysis. (DOCX)
