# Supplementary material for: Phenotypic plasticity influences the success of clonal propagation in industrial pharmaceutical Cannabis sativa
Source: PLoS One. 2019 Mar 18;14(3):e0213434. doi: 10.1371/journal.pone.0213434 (PMC6422331; doi:10.1371/journal.pone.0213434)

**S1 Fig: Detailed description of the data collected from mother plants.** The morphology of mother plants was assessed using a series of characters (Figure S1) including plant height (measured from the first node to the tallest apical meristem), stem diameter (one measurement at the base of the plant and one measurement at 10cm above the base of the plant), the number of apical and lateral meristems, the length of all lateral branches derived from the shortest and the tallest upright branches, the length of all internodes on the tallest and shortest upright branches, the width of the three largest fan leaf blades at the widest point, the length of the petiole from the three largest fan leaves, and the width of the widest point of leaflets of these same three leaves.


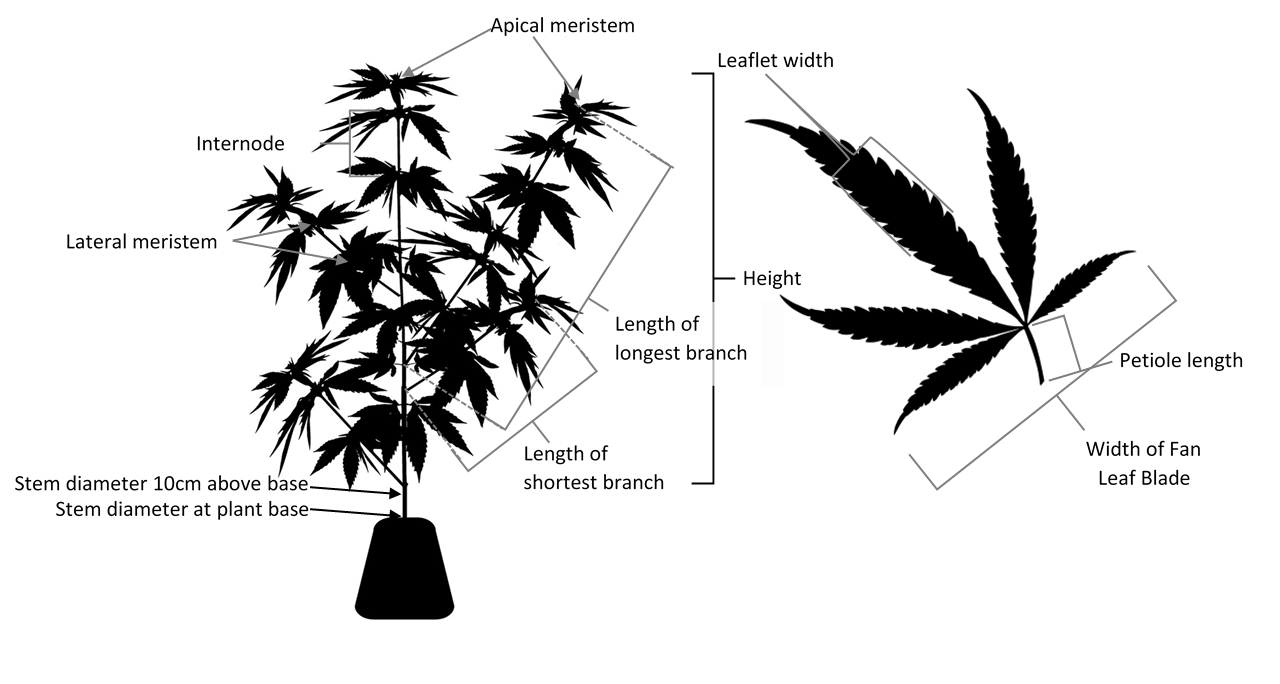

Supplement: S1 Fig — (DOCX) [file pone.0213434.s002.docx]
