# Supplementary material for: Phenotypic plasticity influences the success of clonal propagation in industrial pharmaceutical Cannabis sativa
Source: PLoS One. 2019 Mar 18;14(3):e0213434. doi: 10.1371/journal.pone.0213434 (PMC6422331; doi:10.1371/journal.pone.0213434)
Supplement: S2 Fig — (DOCX) [file pone.0213434.s003.docx]

**S2 Fig: Detailed description of the data collected from stem cuttings.** Cuttings were treated according to the corporate standard operating procedures (using rooting hormone and soil plugs) and were monitored daily for signs of root growth (Figure S2). When roots were detected, the date was recorded. The image below depicts a typical root morphology on the day roots were first detected.
