# Supplementary material for: Phenotypic plasticity influences the success of clonal propagation in industrial pharmaceutical Cannabis sativa
Source: PLoS One. 2019 Mar 18;14(3):e0213434. doi: 10.1371/journal.pone.0213434 (PMC6422331; doi:10.1371/journal.pone.0213434)
Supplement: S1 Code — (DOCX) [file pone.0213434.s004.docx]

**S1 Code. R code for binomial analysis.**

##Open the R library

> library(stats)

##Open the file

>Cloning<-read.csv("Cloning.csv", header=T)

##identify experimental variables as factors for the statistical model – Cultivar, Light, Cutting, and Wound are fixed factors

> Cultivar<-as.factor(Clone$Cultivar)

> Light<-as.factor(Clone$Treatment)

> Cutting<-as.factor(Clone$`Cutting Tool`)

> Wound<-as.factor(Clone$Wounding)

## Tent, Tray, and Position are considered random effects

> Tent<-as.factor(Clone$Tent)

> Tray<-as.factor(Clone$Tray)

> Position<-as.factor(Clone$Position_1)

> NumFan<-as.numeric(Clone$Fanleaves)

##conduct the general linear model using a binomial logistic regression approach

> RootedModel<-glm(Rooted~Light+Cultivar+Cutting+Wound+NumFan+(1|Position)+(1|tent within lighting)+(1|Tray within tent),family=binomial(link="logit"), data=Clone)

##display numerical results of the model

> summary(RootedModel)

##plot the residuals of the model and associated tests

> plot(RootedModel)

##plot relationship of cultivar or effect of wounding treatment (y-axis) vs whether it rooted or not (x-axis)

> plot(Cultivar~Rooted, data=Cloning)

> plot(Wound~Rooted, data=Cloning)

##test the significance of factors in predicting whether the plants rooted or not.

> anova(RootedModel, test="Chisq")
